# *Moringa oleifera* L *.* Nanosuspension Extract Administration Affects Heat Shock Protein-10 and -70 under Orthodontics Mechanical Force In Vivo

**DOI:** 10.1055/s-0044-1791937

**Published:** 2025-01-09

**Authors:** Ari Triwardhani, Alida Alida, Ervina Restiwulan Winoto, Adya Pramusita, Nurul Aisyah Rizki Putranti, Kristian Satrio Ariadi, Okso Brillian Pribadi, Amelia Aisyiah Anwar, Alqomariyah Eka Purnamasari, Rifqah Ananda Mappananrang, Putri Cahaya Situmorang, Wibi Riawan, Tengku Natasha Eleena binti Tengku Ahmad Noor, Albertus Putera Nugraha, Alexander Patera Nugraha

**Affiliations:** 1 Department of Orthodontic, Faculty of Dental Medicine, Universitas Airlangga, Surabaya, Indonesia; 2 Faculty of Dental Medicine, Universitas Airlangga, Surabaya, Indonesia; 3 Department of Biology, Faculty of Mathematics and Natural Sciences, Universitas Sumatera Utara, Medan, Indonesia; 4 Department of Molecular Biochemistry, Faculty of Medicine, Universitas Brawijaya, Malang, East Java, Indonesia; 5 Membership of Faculty of Dental Surgery, Royal Collage of Surgeon, Edinburgh University, United Kingdom; 6 Faculty of Medicine, Universitas Airlangga, Surabaya, East Java, Indonesia

**Keywords:** dentistry, heat shock proteins, medicine, *Moringa oleifera*, orthodontics

## Abstract

**Objective** The mechanical stimulation known as orthodontic mechanical force (OMF) causes biological reactions in orthodontic tooth movement (OTM). Heat shock protein-70 (HSP-70) needs pro-inflammatory cytokines to trigger bone resorption in OTM; nevertheless, heat shock protein-10 (HSP-10), a “Alarmin” cytokine, should control these pro-inflammatory cytokines to get the best alveolar bone remodeling (ABR). *Moringa oleifera* L. nanosuspension extract (MONE) has anti-inflammatory, antioxidant, and ABR-stimulating properties. The aim of the study was to examine *in vivo* HSP-10 and HSP-70 expressions under OMF following MONE application in Wistar rats ( *Rattus norvegicus* ).

**Material and Methods** A total of 36 Wistar rats ( *R. norvegicus* ) were split up into eight groups: one for treatment (OMF + MONE) and one for control (OMF + MONE administration for days 1, 7, 14, and 21). By employing nickel–titanium coil springs and using 10 g of light force per millimeter to implant the orthodontic device, the OMF was completed. According to the day of observation, all of the samples were sacrificed. To perform an immunohistochemistry investigation, the premaxilla of the sample was isolated. Tukey's Honest Significant Different (HSD) test ( *p* < 0.05) was performed after an Analysis of Variance (ANOVA) analysis of the data.

**Results** In both the OMF and MONE groups, HSP-70 peaked on day 14 and began to fall on day 21. HSP-10 peaked on day 21, but along with MONE, it also began to progressively decline on days 14 and 21, with significant differences ( *p* < 0.05).

**Conclusion** According to immunohistochemistry evidence, postadministration of MONE markedly elevated HSP-10 but lowered HSP-70 expression in the alveolar bone of Wistar rats under OMF.

## Introduction

An imperfection in the alignment of teeth or their connection during occlusion is known as malocclusion. Malocclusion affects 56% of both males and females globally. With a malocclusion incidence of 81%, Africa leads the world in this regard, followed by Europe (72%), America (53%), and Asia (48%). Individuals who have malocclusion will have difficulty in speaking, swallowing, and chewing. Psychosocial self-confidence and dentofacial aesthetics can both be negatively impacted by malocclusion. 1 Using orthodontic mechanical force (OMF), orthodontic therapy can realign teeth that are out of alignment. In the other hand, apical root resorption (ARR), the irreversible loss of hard tissue on the root apex of a tooth, is one of the most unfavorable outcomes of orthodontic treatment. Between 20 and 100% of orthodontic patients get ARR. Resorption can be more than 5 mm, or one-fourth of the root length, but severe ARR is rare, occurring only once in 5% of cases. The quality of life and success of orthodontic therapy for individuals affected by ARR may be compromised due to tooth loss and an unequal crown-to-root ratio in the affected teeth. 2 3

By modifying the periodontium of the affected tooth, the pressure from OMF influences orthodontic tooth movement (OTM). In the alveolar bone, both tension and pressure sides are involved in a remodeling process. While the tension side initiates the apposition process, the compression side will experience resorption. OMF-activated osteoblasts and osteoclasts carry out the processes of bone apposition and resorption. It is crucial to use the best OTM possible during orthodontic treatment to avoid harming alveolar bone, periodontal ligament (PDL), or roots. A regulated OMF that applies no more than 20 to 25 g/cm ^2^ of force to the tooth surface can achieve that state. Necrosis in the periodontium may occur if the force is greater than the pressure. 2 3 When a forceful stimulus is applied, the body releases an inflammatory response that causes a localized synthesis and release of multiple molecules, such as growth factors, neurotransmitters, cytokines, colony-stimulating factors, and metabolites of arachidonic acid, which can compromise the nuclear integrity of PDL cells. 4

To preserve a cell's life, heat shock protein (HSP), which is ubiquitous in cells, protects them and aids in their recovery from a variety of stressors. By promoting protein folding and stabilization, enabling transmembrane protein transport, and guaranteeing cell survival under various stress conditions, heat shock proteins-70 (HSP-70s) are molecular chaperones that contribute to the maintenance of cellular homeostasis. OMF increases the concentration of HSP-70 in gingival crevicular fluid (GCF) by causing physiological stress, cell damage, and necrosis in the compressed region through ischemia and hypoxia. A prior work that tracked HSP-70 expression in GCF under orthodontic pressures for 30 days revealed that HSP-70 expression peaked on day thirty. 5 6 Furthermore, heat shock protein-10 (HSP-10) functions as a cochaperone for heat shock protein-60 (HSP-60) and has the capacity to increase Treg activity, which inhibits bone resorption. By activating transcription factors such as runt-related transcription factor 2 (Runx2), alkaline phosphatase (ALP), and osteocalcin (OCN), HSP-10 also aids in the process of osteogenesis. 7 8 9

To achieve homeostasis in the biomechanical and biological mechanism during the tension–pressure phase of the orthodontic treatment and enhance patient satisfaction, the concentration of HSP-70 and HSP-10 during OTM must be regulated. Flavonoids, phenolic acids, and carotenoid compounds are among the several naturally occurring anti-inflammatory and antioxidant compounds found in *Moringa oleifera* L. (MO). These substances also include multivitamins, minerals, calcium, and potassium. The administration of MO extract at 5, 10, and 20% concentration was shown to be able to raise the osteoblast cell count and decrease the osteoclast cell count necessary for alveolar bone regeneration during OTM, according to previous research on MO and OTM. 10 Encapsulation is one of the advanced methods that has recently gained interest in the food sector. The bioactive compounds of MO can be encapsulated for optimal delivery within the human stomach or intestinal tract to produce pleasant, safe, and affordable foods. 11

There is currently insufficient data about how *M. oleifera* L. nanosuspension extract (MONE) affects HSP-10 and HSP-70 during OMF in vivo *.* Furthermore, MONE postadministration during OMF in vivo may have an impact on HSP-10 and HSP-70 expression, according to the study's premise. This in vivo study uses Wistar rats ( *Rattus norvegicus* ) as an animal model to investigate the role of MONE's antioxidant capacity in the stabilization mechanism of HSP-70 and HSP-10 expression under OMF in orthodontic treatment.

## Material and Methods

### Study Design

The laboratory experimental study with a posttest-only control group design was performed. This study protocol was obtained permission from health ethical committee Faculty of Dental Medicine, Universitas Airlangga, Surabaya, Indonesia.

#### *Moringa oleifera* Nanosuspension Extract Preparation

Fresh MO leaves were collected from Puri Kelorina Village, Ngawenombo, Kunduran, Blora, Central Java, 58255, Indonesia, which was registered by the Ministry of Agriculture, Republic of Indonesia, for use in this research. The precise GPS location for where the MO leaves were collected is https://goo.gl/maps/nVmDrneWp7BWstPT9 . The leaves were stored and washed with water, then dried and cut simply. Using ethanol 1:2 (w/v), 350 g MO was macerated and filtered. A vacuum rotary evaporator was utilized for filtrate evaporation at 4°C. The thick extract was desalted and combined with ethanol, resulting in salt settling. This procedure was repeated until the white tint, indicating salt in the solvent, was no longer visible. The preparation of MONE was performed by adding 10 mL of hot aquadest into the mortar, then adding 1 g of carboxymethyl cellulose (CMC) Na, and waiting for 15 minutes. Next, it was stirred until it became a gel mass. Then, 1 g of nipagin solution dissolved in 10 mL of distilled water was added. The extract solution (1 and 2 g), which had been dissolved in 20 mL of 96% ethanol, was then added to the mortar and stirred until homogeneous. After adding 100 g of distilled water, Turax was used for nanosuspension for 10 minutes. The preparation was stirred at 1,400 rpm for 90 minutes.

### Orthodontic Mechanical Force Animal Model

The simple blind random sampling was done. Thus, the sample size was determined by using Lameshow's minimum sample size formula. The sample consisted of 36 healthy Wistar rats ( *R. norvegicus* ), around 16 to 20 weeks old with weights between 200 and 250 g, selected blind-randomly into control and treatment groups, which were then divided into eight groups. OMF was installed by using nickel–titanium close coil spring with 8 mm length (American Orthodontics Corp., Sheboygan, Wisconsin, United States), placed between the incisor and maxillary molars to move the molar mesially with 10 g force per millimeter and measured by using a tension gauge (American Orthodontics Corp., Sheboygan, Wisconsin, United States). The fixed orthodontic appliance was fixed by applying 0.07 stainless steel ligature wire (American Orthodontics Corp., Sheboygan, Wisconsin, United States). Next, the administration of MONE was done by means of microneedle syringe (Hamilton Company, Reno, Nevada, United States) intrasulcular in the gingiva with single dose of 10 μL per day for 1, 7, 14, and 21 days.

All samples were sacrificed 1, 7, 14, and 21 days, respectively, by rodent anesthesia (60 mg/bodyweight of ketamine and 3 mg/bodyweight of xylazine) (Sigma-Aldrich, Merck KGaA, Darmstadt, Germany). Rat's premaxillae were dissected and placed in 10% formalin (OneMed, Surabaya, Indonesia) for 4 days then premaxillae were decalcified for 1 month by using ethylenediaminetetraacetic acid (EDTA) (OneMed, Surabaya, Indonesia).

### Immunohistochemical Assay

The staining conditions for each antibody were adjusted. Each tissue was prepared by removing paraffin and rehydrating it. Retrieving antigens was done by heating the sample in 0.01 M citrate buffer using a domestic microwave oven at full power (750 W) for 15 minutes. Endogenous peroxidase activity was blocked using a solution of methanol containing 3% H _2_ O _2_ by applying it to tissue for 15 minutes.

The slides were then treated with primary antibodies: anti-HSP-10 (HSP 10 Antibody (D-8): sc-376313, Santa Cruz Biotechnology, Inc., Dallas, Texas, United States) at a 1:500 dilution and anti-HSP-70 (Anti-Heat Shock Protein 70 (HSP70) antibody, Mouse monoclonal, SAB4200714, Abcam, Sigma-Aldrich, Burlington, Massachusetts, United States) at a 1:500 dilution. Then, the slides were incubated at 4°C overnight. Afterward, all slides were washed three times with phosphate-buffered saline (PBS) for 5 minutes. Then visualization signals were generated using a stain kit (DAB) (Sigma-Aldrich, Merck KGaA, Darmstadt, Germany). Following this, all slides were counterstained with Hematoxylin-Eosin (Sigma-Aldrich, Merck KGaA, Darmstadt, Germany).

Immunohistochemical staining was assessed independently by Wibi Riawan and Alexander Patera Nugraha, both of which were blinded to the clinicopathological data. This assessment was done under a light microscope at 400× and 1000× magnification. The cytoplasm was the location of HSP-10 and HSP-70 proteins. This is in line with the principle of antibody interaction with a specific target protein located in the tissue, as the interaction detected may be visible due to a chromogenic agent thus appearing in the form of a specific color as graded by the density, whereas the darker the density refers to higher levels of target proteins on the specific site.

### Data Analysis

The data were analyzed by Statistical Package for the Social Sciences 20.0 software (SPSS for Windows, Chicago, Illinois, United States). Descriptive statistics were given using mean or average ± standard deviation (SD). One-way analysis of variance (ANOVA) and Tukey's Honest Significant Difference (HSD) ( *p* < 0.05) were implemented based on Saphiro–Wilk and Levene's test ( *p* > 0.05).

## Results

**Fig. 1:**
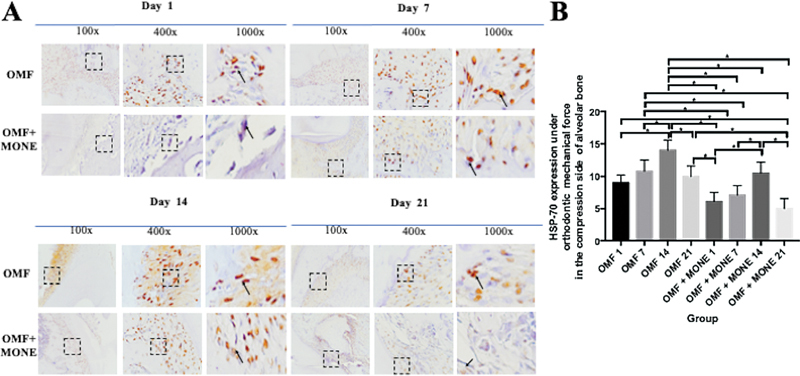
The comparison of IHC results shows an HSP-70 positive expression in the fibroblast of the alveolar bone of Wistar rats (Rattus norvegicus) during OMF with 100x, 400x, and 1000x magnification by light microscope. ( **A** ) The black arrow pointed to the positive expression of HSP-70 in the compression side in each group. ( **B** ) Represent the positive number of HSP-70 expression in the compression side between groups. Data are expressed as mean±SD (n .7), significant at p < 0.05. OMF1 : positive control group with OMF and PBS administration for 1 days; OMF7 : positive control group with OMF and PBS administration for 7 days; OMF14 : positive control group with OMF and PBS administration for 14 days; OMF21 : positive control group with OMF and PBS administration for 21 days; OMF+MONE1: group with OMF for 14 days and MONE administration from day 1; OMF+MONE7: group with OMF for 14 days and MONE administration from day 1 to day 7; OMF+MONE14: group with OMF for 14 days and MONE administration from day 1 to day 14; OMF+MONE21: group with OMF for 14 days and MONE administration from day 1 to day 21; HSP-70, heat shock protein-70; IHC, immunohistochemical; MONE, Moringa oleifera L. nanosuspension extract; OMF, orthodontic mechanical force.

**Fig. 2:**
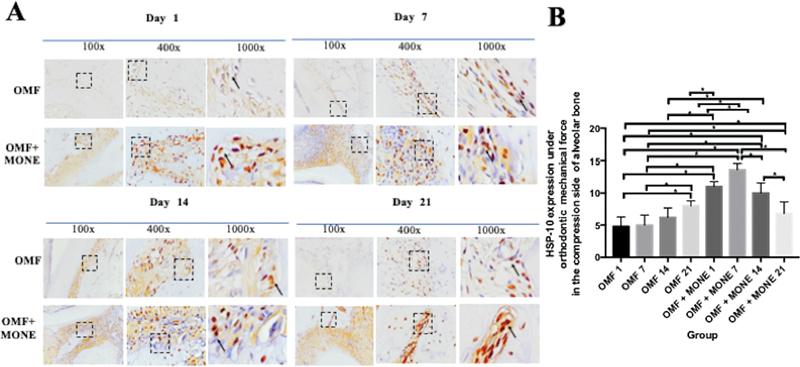
The comparison of IHC results shows an HSP-10 positive expression in the fibroblast of the alveolar bone of Wistar rats (Rattus norvegicus) during OMF with 100x, 400x, and 1000x magnification by light microscope. ( **A** ) The black arrow pointed to the positive expression of HSP-10 in the compression side in each group. ( **B** ) Represent the positive number of HSP-10 expression in the compression side between groups. Data are expressed as mean±SD (n .7), significant at p < 0.05. OMF1 : positive control group with OMF and PBS administration for 1 days; OMF7 : positive control group with OMF and PBS administration for 7 days; OMF14 : positive control group with OMF and PBS administration for 14 days; OMF21 : positive control group with OMF and PBS administration for 21 days; OMF+MONE1: group with OMF for 14 days and MONE administration from day 1; OMF+MONE7: group with OMF for 14 days and MONE administration from day 1 to day 7; OMF+MONE14: group with OMF for 14 days and MONE administration from day 1 to day 14; OMF+MONE21: group with OMF for 14 days and MONE administration from day 1 to day 21; HSP-10, heat shock protein-10; IHC, immunohistochemical; MONE, Moringa oleifera L. nanosuspension extract; OMF, orthodontic mechanical force.

HSP-70 expressed positively in brown color in the fibroblast’s cytoplasm in the compression side of alveolar bone day 1, 7, 14, and 21 ( Fig. 1A ). The expression of HSP-70 reached its peak on day 14 and started to decline on day 21 in both the OMF andMONE groups ( Fig. 1B ) with significant difference between groups (p. 0.001; p<0.05). HSP-70 expressed positively in brown color in the Fibroblast’s cytoplasm in the compression side of alveolar bone day 1, 7, 14, and 21 21 ( Fig. 2A ). On the other hand, HSP-10 expression reached its highest on day 21, but in addition to MONE, it was starting to incline gradually on days 14 and 21 ( Fig. 2B ) with significant difference between groups (p.0.001; p<0.05).

## Discussion

In the presence of OMF, OTM is a continuous, balanced process that is typified by bone resorption and deposition in regions of compression and stress. Adsorption occurs on the pressure side and localization occurs on the stress side according to OTM's pressure stress theory. Whereas bone production occurs in areas of PDL tension, OMF causes alveolar bone loss in areas of PDL stress. 3 12

Coordinated processes of bone resorption and deposition are activated by OTM and promoted by OMF. 13 It may be schematically split into four phases based on the pace at which teeth move: the initial phase, lag phase, accelerating phase, and linear phase. Early on, the bone is somewhat bent and the teeth shift on the stretch side due to OMF compressing the PDL. Localized necrotic areas formed in the PDL and the lamina dura was mechanically blocked as a result of the lag phase (arrest). Phagocytic cells and activated osteoblasts from the alveolar bone marrow side eliminate these regions. The state called as undermining resorption will develop if surpassing OMF is continuous. When mechanical strength is at its peak, the acceleration phase happens, causing the teeth to move more quickly. The linear phase of motion always comes next if the force is kept constant. 14

Alveolar bone remodeling (ABR) is induced by mechanical stress in two ways that lead to OTM. 15 First, the force is conveyed from the PDL to the alveolar bone by the presence of OMF that is delivered to the tooth. This results in a mild, reversible lesion to the periodontium that supports the tooth. 16 17 Selective ABR at opposing sites around the teeth is a symptom of this disorder. The terms “tension” and “compression” are used to describe these discrete locations, depending on the dominating forces and the overall tissue reaction. The PDL fibers that connect the tooth to the bone experience strain on the tension side as a result of the displacement of the dental root.

The PDL on the opposing side of the tooth experiences a compressive force when the tooth moves in the direction of the force, squeezing it closer to the bone. The PDL fibers that bind the tooth to the bone are not loaded in this region. As a result, when mechanical stresses are applied to cells, a biological reaction occurs that results in bone formation at the tension site and resorption at the compression site. It is interesting to note that this is different from bone, where pressure causes new bone to grow. 18

Second, aseptic inflammation of the PDL at the tension region is the cause of OTM. Aseptic inflammation is the term used to characterize the biological reaction to OTM, which is mediated by a variety of inflammatory cytokines, neuropeptides, and vasoactive substances. The generation of chemoattractant and the recruitment of leukocytes are stimulated by localized damage to the PDL caused by inflammatory processes in the pressure region. Injured tissue in the PDL gap is removed by leukocytes. Osteoclast precursors are enlisted concurrently to promote bone resorption. On the other hand, the inflammatory response on the compression side may be very different from that on the other side, and the production of distinct growth factors and cytokines would favor a predominance of bone-forming activity. 19

The three phases of the biological reaction to tooth movement are as follows: (1) teeth movement resulting from PDL pressure (gradual pressure of PDL that may last ∼4–7 d); (2) hyalinization period (cell death due to lack of blood supply in areas of pressure on PDL with very little or no tooth movement takes place for 10–20 d); and (3) tooth movement resulting from undermining resorption, which is largely mediated by the presence of oxygen, caused by the reopening of the capillary network. 12 In relation to this situation, overcoming a stable OTM in fixed orthodontics requires achieving equilibrium in ABR.

A vital component of the HSP family, HSP-70 is essential for maintaining cell viability under a variety of stressors. Orthodontic therapy puts strain on periodontal tissue, which causes ischemia and hypoxia-induced necrosis in the compressed region and cell death. 20 According to research by Bozkaya et al, GCF exhibits an increase in HSP-70 expression in response to these stimuli. OTM-related cytoprotection and host immunological defense are linked to the increased concentration of HSP-70 in GCF. 5 To evaluate HSP-70 and toll-like receptor 4 (TLR-4) expression in GCF, Bozkaya et al performed a study beginning at baseline, continuing until 1 hour after the force application (T2), and continuing until 4, 7, 14, and 30 days after the force application (T3, T4, T5, T6). More tooth movement is associated with more injured cells in the compressed PDL, which helps explain the gradual increase in HSP-70 expression from T2 to T6. 5

HSP-70 is an endogenous ligand for TLR-4 and has been found in the literature as a danger-associated molecular pattern (DAMP). By interacting with TLR-4, it can trigger many signaling pathways, including nuclear factor kappa-light-chain-enhancer of activated B cells (NF-κ-β), which in turn increases proinflammatory cytokines like interleukin-1β (IL-1β). One of the most important proinflammatory cytokine of OTM that osteoclasts release is IL-1β. 19 20 Bozkaya et al found that while IL-1β started to grow at T3, which coincided with the peak of TLR-4, HSP-70 reached its maximum level at T6. Up to T6, IL-1β expression was still rising. Under those circumstances, it may be concluded that HSP-70 functions as a DAMP for the TLR-4 signaling pathway, which may be involved in the synthesis of IL-1β in response to mechanical stress during OTM. 5

A surge in HSP-70 was also noted in 24 hours after mechanically stretching the Malassez porcine epithelial rest cells. Afterwards, they observed that HSP-70 expression declined with time. According to research by Thulasidharan et al, the sustained ribosomal activity can be the reason for the enhanced expression of HSP-70 caused by OMF stress after 28 days as compared with the control group. As evidenced, the decrease in HSP-70 expression may represent a rate-limiting event, resulting in a drop in HSP-70 synthesis once the tissue reaches homeostasis. 4

As a cochaperon of HSP-60, HSP-10 aids in the folding and binding of freshly synthesized proteins as they move from the cytoplasm into the mitochondria. ^20^ It has been demonstrated that HSP-10 is the extracellular homolog of chaperonin 10 (CPN-10), an HSP. CPN-10 is a strong promoter of bone resorption and osteoclast recruitment. Because type I collagen production is markedly enhanced when osteoblasts are treated with CPN-10, HSP-10 has also been proposed to have a function in bone collagen synthesis and contribute to the process of bone resorption. 21 It suggested, however, indirectly that HSP-10 could be involved in the process of ABR during the OTM phase.

Under normal conditions, the first reaction to OMF given to the PDL occurs during the first 2 to 4 hours, is followed by an inflow of osteoclasts to the compression sites, and lasts for 4 to 7 days. When using fixed orthodontics, OTM typically requires 2 to 14 days to induce bone resorption and counteract tooth movement. However, under abnormal conditions, excessive bone resorption can lead to undermining resorption, which can cause massive tooth movement as indicated by hyalinization of tissue. The immune system then needs to remove this tissue, which is typically completed within the lag phase, which is 4 to 20 days. 5 22 23

As OMF is considered a stressor and triggers an inflammatory cascade by upregulating the expression of HSP-70. 5 24 25 It is normally this that initiates bone resorption on the compression sites. HSP-70 will then attach to TLR-4, which will cause an increase in Iκα kinase (IKKα) and IκB kinase (IKKβ), which will both activate NF-kβ, the main proinflammatory cascade. The modification of many proinflammatory cytokines, including matrix metalloproteinase-2/9 (MMP-2/9), Tumor necrosis factor alpha (TNF-α), and IL-1β/6, occurs during activation of NF-kβ. 26 27 TNF-α and IL-1β/6 then modulate osteoclastogenesis in response to the MMP-2/9 family of proteolytic enzymes breaking down the extracellular matrix (ECM) of bone components. 28 29 30 These proteins increase the expression of Macrophage colony-stimulating factor (M-CSF), thrombin receptor activator peptide 6 (TRAP6), and nuclear factor of activated T-cells cytoplasmic 1 (NFATc1). These expressions are reflected in the development of osteoclast precursor cell megakaryocytic colony-forming cells (CFU-M) into preosteoclast and eventually mature osteoclast. 30 31 This sequence should occur within the normal work frame because disruption or prolonged activation of osteoclastogenesis, as well as prolonged elevation of HSP-70 levels, may increase the likelihood of undermining resorption. This is related to normal circumstances, which typically involve tooth movement lasting up to 14 days, after which the process of bone resorption should begin to decline. To treat this illness, it is necessary to restore equilibrium by modifying a protein like HSP-70 that has opposing properties to halt the inflammatory process. 20

As a cochaperone for HSP-60, HSP-10 is a protein that can trigger Treg activity. This leads to the downregulation of Receptor activator of nuclear factor kappa-Β ligand (RANKL), NFATc1, and TRAP6, which inhibits osteoclastogenesis and subsequent bone resorption. Conversely, Treg may activate the production of several osteogenic transcription factors, such as OCN, ALP, and Runx2. To promote the creation of new bone, these proteins aid in the proliferation and differentiation of osteoblast precursors into preosteoblasts and mature osteoblasts. 7 8 9 In light of this, it is imperative to maintain precise levels of HSP-10 and -70 to accomplish such ideal ABR.

One of the members of the Moringaceae family is MO, often known as “kelor” in Indonesian. The active ingredients in MO include vitamins, alkaloids, glucosinolates, carotenoids, polyphenols, phenolic acids, flavonoids, isothiocyanates, tannins, saponins, and oxalates. Myricetin, quercetin, and kaempferol were the primary flavonoids identified in MO leaves, with amounts of 5.8, 0.207, and 7.57 mg/g, respectively. The polyphenols (kaempferol and tannin) in MO have antioxidant qualities that can decrease osteoclastogenesis, modify osteogenesis, and suppress reactive oxygen species. The inclusion of flavonoids, such as saponin and quercetin, which have anti-inflammatory, antioxidant, and osteogenesis-modulating properties, lends credence to this. These proteins function by decreasing the production of TNF-α, IL-1β/6, and Prostaglandin E2 (PGE2), suppressing NF-κB activation, and controlling the expression of Inducible Nitric Oxide Synthase (iNOS), Interferon‐gamma (IFN-γ), and C-reactive protein. Furthermore, they preserve the health of osteoblasts and osteocytes by regulating the Receptor activator of nuclear factor kappa-Β ligand-osteoprotegerin (RANKL–OPG) complex. Lastly, they lessen the differentiation and activity of osteoclasts due to the downregulation of NF-kβ. 10 32 33 34 35

This study shows that MO extract in the form of nanosuspension (MONE) can control HSP-10 and -70 expression. According to immunohistochemistry documentation, MONE functions by suppressing the expression of HSP-70, whose level peaked on day 14 and began to fall on day 21. Conversely, in contrast to the control group, the level of HSP decreased more sharply on day 21 in addition to MONE. Given that MONE lowers HSP-70 to quicken the inflammatory phase and avoid excessive bone resorption, this suggests that MONE may regulate HSP-70 expression. This disease may result from HSP-70's primary target, TLR-4, disrupting its persistent sensitivity. This results in the inhibition of the interaction between MyD88, a downstream protein, and TRAF6, which is the tumor necrosis factor receptor-associated factor 6. Thus, this situation leads to the inactivation of I-κB (IκB) and mitogen-activated protein kinase (MAPK), which in turn causes NF-κB downregulation. Additionally, it disturbs the activation of transforming growth factor-β-activated kinase 1 (TAK-1) and transforming binding protein 2 and 3 (TAB2/3). 26 27 29 36 Proinflammatory cytokines such as TNF-α and IL-1β/6 are impacted by this. In addition to modulating Tissue inhibitor of metalloproteinases 1 (TIMP-1) by MONE's bioactive chemicals, downregulating both proteins inhibits MMP-2/9, an enzyme that causes the breakdown of ECM. This might stop more bone loss. 36 37 38

On the other side, M-CSF and RANKL signaling are reduced when TNF-α is downregulated. Because of this, the adaptor protein TRF-6 is not absorbed, which inhibits NFATc-1 and stops osteoclasts from producing maturation. By inhibiting IL-1β, PGE2 production is decreased, which in turn causes Receptor Activator of Nuclear factor Kappa B (RANK) to be downregulated. This prevents osteoclast cells from differentiating, activating, and surviving. Because RANK is downregulated when IL-6 is inhibited, preosteoclast sensitivity to RANKL stimulation is reduced. 39 40

Furthermore, this study demonstrates a significant improvement in MONE's capacity to regulate HSP-10 levels, whereas HSP-10 expression peaked on day 7 and then progressively decreased on days 14 and 21, but it was still greater than in the control group. Because this state inhibits excessive inflammation, osteogenesis is accelerated and further osteoclastogenesis is inhibited. In addition to the change in HSP-70 level brought on by MONE administration, this may also disrupt the equilibrium of both proteins, resulting in an ideal ABR. This scenario is conceivable because Treg is still being modulated, which helps inhibit the RANKL activity and halt bone deterioration. This is made feasible by the fact that HSP-10 levels are beginning to rise. However, MONE that has been enhanced with different flavonoids and alkaloids can alter Vascular endothelial growth factor (VEGF), which is a protein that affects angiogenesis. In addition to inducing the expression of fibroblast growth factor 2 (FGF-2), which results in the formation of fibroblasts to repair damaged periodontal tissue, VEGF works by inducing the formation of new blood vessels to maintain oxygen and nutrition transport and enable an optimal regeneration of periodontium. 41 42

By influencing the production of bone morphogenetic protein 2 (BMP-2), VEGF and FGF-2 are essential in promoting osteogenesis. This is demonstrated by the activation of the suppressor of mothers against decapentaplegic 1/5/8 (Smad-1/5/8). 43 44 ALP, Osteorix (Osx), Runx2, and other osteogenic transcription factors are modulated in this way. Runx2 is a versatile transcription factor that plays a crucial role during osteogenic development. It has the ability to influence the transcription of other genes associated with osteoblasts, such as OCN and collagen type I α1 chain (COLL1A1). Runx2 stimulates the differentiation of mesenchymal cells into osteoprogenitor cells. At this point, osteoprogenitor cells proliferate into juvenile osteoblasts with the aid of Runx 2, Osx, and distal-less homeobox 5 (Dl × 5). ALP, COLL1A1, and bone sialoprotein are among the ECM proteins that aid in the maturation of immature osteoblasts into mature osteoblasts that are abundant in OCN and osteopontin. At this stage, the mineralization process is aided by proteins and ALP, which provide an alkaline environment in osteoid tissue to facilitate the easy deposit of Ca ^2+^ . The development of mature bone tissue made up of osteocytes signaled the conclusion of the mineralization process. 32 45 46 47 48 In relation to this process, optimal bone regeneration is facilitated by the modulation of HSP-10 and -70 levels, as demonstrated by MONE's exceptional capacity. This presents a significant opportunity to surpass the favorable prediction for the success rate of fixed orthodontics application in clinical practice. 49 50 51 52

However, there are several drawbacks to this study, including the small number of biomolecular markers that were examined and the methodologies used for the assessment. To clarify the precise mechanism by which MONE alters ABR under OMF in vivo, more research is still required.

## Conclusion

The immunohistochemical results of this study show that, in the alveolar bone of Wistar rats ( *R. norvegicus* ), postadministration of MONE significantly raises HSP-10 level but lowers HSP-70 expression with significantly different. Nonetheless, more research is required to examine additional biomolecular indicators connected to the remodeling of alveolar bone during orthodontic mechanical stress using diverse methodologies.
